# Aryl hydrocarbon receptor activation-mediated vascular toxicity of ambient fine particulate matter: contribution of polycyclic aromatic hydrocarbons and osteopontin as a biomarker

**DOI:** 10.1186/s12989-022-00482-x

**Published:** 2022-06-23

**Authors:** Chia-Chi Ho, Wei-Te Wu, Yi-Jun Lin, Chen-Yi Weng, Ming-Hsien Tsai, Hui-Ti Tsai, Yu-Cheng Chen, Shaw-Fang Yet, Pinpin Lin

**Affiliations:** 1 National Institute of Environmental Health Sciences, National Health Research Institutes, 35, Keyan Road, Zhunan Town, Miaoli County, 350 Taiwan; 2 Institute of Food Safety and Health Risk Assessment, National Yang Ming Chiao Tung University, 155, Sec. 2, Linong Street, Taipei, 112 Taiwan; 3 Institute of Cellular and System Medicine, National Health Research Institutes, 35, Keyan Road, Zhunan Town, Miaoli County, 350 Taiwan

**Keywords:** Ambient particulate matter, VSMC migration, Osteopontin, Aryl hydrocarbon receptor, Polycyclic aromatic hydrocarbons, Biomarker

## Abstract

**Background:**

Exposure to ambient fine particulate matter (PM_2.5_) is associated with vascular diseases. Polycyclic aromatic hydrocarbons (PAHs) in PM_2.5_ are highly hazardous; however, the contribution of PM_2.5_-bound PAHs to PM_2.5_-associated vascular diseases remains unclear. The ToxCast high-throughput in vitro screening database indicates that some PM_2.5_-bound PAHs activate the aryl hydrocarbon receptor (AhR). The present study investigated whether the AhR pathway is involved in the mechanism of PM_2.5_-induced vascular toxicity, identified the PAH in PM_2.5_ that was the major contributor of AhR activation, and identified a biomarker for vascular toxicity of PM_2.5_-bound PAHs.

**Results:**

Treatment of vascular smooth muscle cells (VMSCs) with an AhR antagonist inhibited the PM_2.5_-induced increase in the cell migration ability; NF-κB activity; and expression of cytochrome P450 1A1 (CYP1A1), 1B1 (CYP1B1), interleukin-6 (IL-6), and osteopontin (OPN). Most PM_2.5_-bound PAHs were extracted into the organic fraction, which drastically enhanced VSMC migration and increased mRNA levels of CYP1A1, CYP1B1, IL-6, and OPN. However, the inorganic fraction of PM_2.5_ moderately enhanced VSMC migration and only increased IL-6 mRNA levels. PM_2.5_ increased IL-6 secretion through NF-κB activation; however, PM_2.5_ and its organic extract increased OPN secretion in a CYP1B1-dependent manner. Inhibiting CYP1B1 activity and silencing OPN expression prevented the increase in VSMC migration ability caused by PM_2.5_ and its organic extract. The AhR activation potencies of seven PM_2.5_-bound PAHs, reported in the ToxCast database, were strongly correlated with their capabilities of enhancing the migration ability of VSMCs. Benzo(k)fluoranthene (BkF) contributed the most to the AhR agonistic activity of ambient PM_2.5_-bound PAHs. The association between PM_2.5_-induced vascular toxicity, AhR activity, and OPN secretion was further verified in mice; PM_2.5_-induced intimal hyperplasia in pulmonary small arteries and OPN secretion were alleviated in mice with low AhR affinity. Finally, urinary concentrations of 1-hydroxypyrene, a major PAH metabolite, were positively correlated with plasma OPN levels in healthy humans.

**Conclusions:**

The present study offers in vitro, animal, and human evidences supporting the importance of AhR activation for PM_2.5_-induced vascular toxicities and that BkF was the major contributor of AhR activation. OPN is an AhR-dependent biomarker of PM_2.5_-induced vascular toxicity. The AhR activation potency may be applied in the risk assessment of vascular toxicity in PAH mixtures.

**Supplementary Information:**

The online version contains supplementary material available at 10.1186/s12989-022-00482-x.

## Introduction

Cardiovascular diseases such as atherosclerosis [1] and pulmonary arterial hypertension are major health effects associated with exposure to ambient fine particulate matter (PM_2.5_) [2, 3]. Epidemiological studies have reported that long-term exposure to ambient PM_2.5_ is associated with increased carotid intima–media thickness, which is an indicator of early atherosclerosis [4, 5]. PM_2.5_ exposure may trigger atherosclerosis by disturbing the functions of endothelial cells in the tunica intima and of vascular smooth muscle cells (VSMCs) in the tunica media [6, 7]. In response to various stimuli, VSMCs can switch from a highly differentiated (contractile) phenotype to a “dedifferentiated” (also termed “synthetic”) phenotype, with an increased proliferative and migratory ability and proinflammatory protein secretion [8]. Exposure to ambient PM_2.5_ causes medial thickening and intimal hyperplasia in pulmonary small arteries of mice [7]. Exposure to ambient PM_2.5_ induced phenotypic changes in VSMCs, including an increase in cell proliferation, migration, and proinflammatory cytokine secretion [7, 9]. Therefore, VSMC phenotypic changes may be a mechanism-based indicator of the risk of PM_2.5_-induced vascular toxicity.

PM_2.5_ is a complex mixture of various chemical components. Some epidemiological studies have demonstrated that concentrations of certain components in PM_2.5_, such as black carbon, organic carbon, sulfates, nitrite, nitrate, vanadium, nickel, iron, and zinc, are associated with cardiovascular effects, mortality, or morbidity [10–13]. In addition to ions and metals, PM_2.5_ contains many polycyclic aromatic hydrocarbons (PAHs) [14, 15]. Although the mass concentrations of PAHs in ambient PM_2.5_ are < 0.1%, some PAHs are highly hazardous. Inhalation exposure to PAHs can have various toxic effects, such as respiratory effects [16], immunological effects, and cancers [17]. Researchers have studied the carcinogenesis of PM_2.5_ or the carcinogenic risk of exposure to ambient PM_2.5_-bound PAHs [18, 19]. However, the contribution of ambient PM_2.5_-bound PAHs to the risk of cardiovascular disease has rarely been investigated.

The assessment of the carcinogenic risks of PAHs is based on relative potency factors (RPFs), which are defined as the cancer potency of different PAHs relative to that of benzo[a]pyrene (BaP) [20]. However, because the mechanisms of carcinogenesis and vascular toxicity are completely different, data of PAH-induced vascular toxicity are lacking, and mechanisms related to PAH-induced vascular toxicity are largely unknown, the RPFs of PAHs cannot be applied to assess the vascular toxicity risk of PAHs. More than 20 PAHs have been identified in ambient PM_2.5_. To assess the cumulative vascular toxicity risk of PM_2.5_-bound PAHs, identifying a common mechanism of vascular toxicity for different PAHs is necessary.

Some PAHs are agonists of the aryl hydrocarbon receptor (AhR), a ligand-activated transcription factor. Typically, some PAHs activate the AhR and subsequently upregulate the gene expression of cytochrome P450 1A1 (CYP1A1) and 1B1 (CYP1B1)[21]. AhR activation mediates some toxicities, such as carcinogenicity and immunotoxicity [22, 23]. The information regarding AhR activation potencies of most PAHs is available in the ToxCast high-throughput screening (HTS) database. The ToxCast program, initiated by US EPA, involved quantitative HTS for approximately 10,000 chemicals with many in vitro bioassays, including bioassays for AhR activation [24]. If AhR activation is proven to be involved in the mechanism of PM_2.5_-induced vascular toxicities, we may use the AhR activation potency to assess the vascular toxicity risk exerted by PM_2.5_-bound PAHs.

We previously demonstrated that cotreatment with an AhR antagonist prevented the ambient PM_2.5_-induced proliferation of VSMCs [25]. Thus, the AhR pathway might be involved in the mechanism of PM_2.5_-inudced phenotypic changes in VSMCs. BaP induces osteopontin (OPN) expression in lung cancer cells, which was repressed by the AhR antagonist [26]. OPN, a pleiotropic cytokine, is expressed in various cells, such as smooth muscle cells, endothelial cells, immune cells, and lung cancer cells [27, 28]. OPN is involved in the pathophysiology of vascular diseases, including atherosclerosis [29], neointimal hyperplasia [30], and pulmonary arterial hypertension [31]. Overexpression of OPN in transgenic mice increased the neointima and medial thickness of the aorta following arterial injury [30]. We previously reported that exposure to ambient PM_2.5_ increased OPN secretion in VSMCs, as well as in mice [9]. Furthermore, exposure to ambient PM_2.5_ was positively correlated with plasma OPN levels in young adults [9]. Therefore, we hypothesized that PM_2.5_-bound PAHs might cause vascular toxicity by activating the AhR pathway. Our results may guide the establishment of a mechanism-based approach for assessment of the cumulative vascular toxicity risk exerted by PM_2.5_-bound PAHs.

## Methods

### Materials

CH-223191 (AhR inhibitor) was purchased from Sigma (St. Louis, MO, USA). PS-1145 (IKK inhibitor) was purchased from Cayman Chemical Company (Ann Arbor, MI, USA). (E)-2,3',4,5'-tetramethoxystilbene (TMB) (CYP1B1 inhibitor) was purchased from MedChemExpress (Monmouth Junction, NJ, USA).

### PM_2.5_ sample collection

PM_2.5_ samples were collected from January to March 2018, in Kaohsiung City, Taiwan, through high-volume impaction by using a Digitel DHA-80 aerosol sampler (Digitel, Hegnau, Switzerland) at 500 L/min. The samples were collected on fiberglass filters coated with polyvinylidene difluoride (Pallflex Fiberfilm TX40HI20; Pall Corporation, New York, NY, USA). Characteristics and chemical components of PM_2.5_ samples collected in 2018 were previously described [25]. All samples were stored at − 20 °C after collection. Before and after each collection, the fiberglass filters were weighed using standard operating procedures in an environmentally controlled room (23 ± 1 °C with a relative humidity of 40% ± 5%) on an analytical balance (AG204 dual-range, Mettler Toledo, Columbus, OH, USA) to determine the amount of PM_2.5_ collected.

### PM_2.5_ extracts

The fiberglass filters used for collecting PM_2.5_ or the blank filter control were wetted with 70% ethanol in a glass measuring beaker and subsequently sonicated for 30 min at room temperature. PM_2.5_ was extracted as previously described [9]. We also weighed the collecting tubes before and after extraction and condensation. The recovery of PM_*2.5*_ extraction was 86.5%. The proportion of the blank fiberglass filter in extracted PM_2.5_ was approximately 5% (weight/weight).

### Preparation of organic and inorganic PM_2.5_ extracts

The organic fractions of PM_2.5_ were extracted with 100 mg of PM_2.5_ and 300 mL of a mixture of acetone and hexane (2:3, v:v) by using the CEM MARS Xpress Microwave Accelerated Reaction System (CEM Corporation, Matthews, NC, USA). The extracted PM_2.5_ was separated from the solvent through centrifugation for 10 min at 1500 × *g*. The supernatant was the organic PM_2.5_ fraction. The residual PM_2.5_ was dried and sonicated in double-distilled water (d_2_H_2_O) for 10 min before being centrifuged for 10 min at 13,500 × *g*. The supernatant was the inorganic (water soluble) PM_2.5_ fraction.

### Animal experiments

B6.D2N*Ahr*^*d*^/J mice were purchased from the Jackson Laboratory (Bar Harbor, ME, USA) and bred at the National Health Research Institutes (NHRI). *Ahr*^*d*^ is naturally present in DBA/2 strains. The B6.D2N*Ahr*^*d*^/J strain has DBA/2 alleles for a portion of proximal chromosome 12 introgressed into the C57BL/6J background. All animal treatments and experimental protocols (NHRI-IACUC-107022-A) were reviewed and approved by the Institutional Animal Care and Use Committee of the NHRI. Five mice were housed of a cage under a 12-h light–dark cycle at 23 ± 1 °C, with a relative humidity of 39–43%. Water and food were provided ad libitum. Male Ahr^d^(B6.D2)and wild-type(B6) mice (eight-weeks-old) were either exposed to d_2_H_2_O as control or 25 μg of PM_2.5_ for 12 weeks. Ten mice (n = 10) from each group were randomly selected for experimentation, and a total of forty mice were used. The mice were exposed to 25 μg PM_2.5_/mouse twice weekly through oropharyngeal aspiration. Bronchoalveolar lavage fluid (BALF) were collected to distinguish different cell types and measure OPN and IL-6 concentration. The lung tissue were collected for histological analysis and immunohistochemistry. For each analysis, the data of each animal in each experimental group were inlcuded.

### Oropharyngeal aspiration

The mice were anesthetized through isoflurane inhalation. While under anesthesia, each mouse was secured on its back on an inclined plane, with its head elevated. The mouth was secured in an open position with a rubber band, and the tongue was held to one side by using forceps to facilitate visualization of the epiglottis. A syringe fitted with a blunt, polished needle (19 gauge, 3 inches long, angled at 45°) was inserted into the mouth of the mouse until it reached its larynx. The sample was then rapidly expelled. The mice were given 30 μL of distilled water or 25 μg of PM_2.5_ per 30 μL of water.

### Preparation and evaluation of the BALF

The mice were sacrificed through overdose of isoflurane inhalation. The whole lung was was dissected out surgically and was lavaged 3 times with 1 mL of saline.

The recovered amount of lavagate was recorded and saved in individually labelled bottles. The total cell numbers in the BALF from the animals were determined with a cell counter (Coulter Inc., Miami, FL,USA). BALF was centrifuged at 800 g × for 15 min using a Shandon Cytospin 4 (Thermo Scientific, Waltham, MA, USA). The cytospin smear was then prepared and Liu’s staining (Tonyar Biotech, Tao Yuan, Taiwan) was performed to distinguish different cell types.

### Histological analysis and immunohistochemistry

We isolated the left lobe and right inferior lobe of the lung and horizontally cross sectioned the middle part of the lobes, which contained secondary bronchi, bronchioles, alveolar ducts and sac. The protions of lung tissues were fixed using 10% neutral buffered formalin for 48 h prior to tissue processing, including dehydration, clearing, and embedding in paraffin. Hematoxylin and eosin (H&E) staining was performed for histopathological examinations according to a previously described protocol [32]. Briefly, paraffin sections were dewaxed and rehydrated, and smooth muscle alpha-actin (SMA) (Sigma-Aldrich A5228, St. Louis, MO, USA), OPN (Proteintech 25725-1-AP,Chicago, Illinois, USA) and IL-6 (Bioss bs-0379R, Woburn, MA, USA) antibodies were applied. For immunohistochemistry, sections were heated in a 0.01 M citrate buffer for 10 or 20 min. Subsequently, specific antibodies were incubated on the sections for 8–16 h at room temperature. The secondary linked antibody and the polymer–peroxidase conjugate (DakoCytomation, Glostrup, Denmark) were then incubated on the sections for 10 min each. The sections were stained using diaminobenzidine (DakoCytomation, Glostrup, Denmark) for detection and with hematoxylin for counterstaining (Muto Pure Chemicals, Tokyo, Japan).

### Cell culture

Primary mouse VSMCs were isolated from mouse aortas and cultured in Dulbecco’s Modified Eagle Medium (GIBCO, Carlsbad, CA, USA) with L-glutamine, sodium bicarbonate, and fetal bovine serum (FBS), as described [33]. Cells at passages 5–8 were used in the following experimental assays and incubated at 37 °C in a humidified condition of 5% CO_2_ and 95% air.

### Quantitative real-time reverse transcription-polymerase chain reaction assays

A total of 3*10^5^ VSMCs in 6 cm dish were treated with vehicle or PM_2.5_ with or without inhibitors in 3 ml of 0.5% FBS media for 48 h. Total RNA was prepared using RNAzol reagent (Life Technologies, Rockville, MD, USA). The cDNA was synthesized using a High-Capacity cDNA Archive Kit (P/N4322171, Applied Biosystems, Foster City, CA, USA). The PCR primers for CYP1A1, CYP1B1, OPN, and glyceraldehyde-3-phosphate dehydrogenase were added to the Assays-on-Demand Gene Expression Assay Mix (Applied Biosystems). Quantitative polymerase chain reaction (qPCR) assays were then conducted using a TaqMan Universal PCR Master Mix (Applied Biosystems) and an ABI StepOnePlus real-time PCR system (Perkin–Elmer, Applied Biosystems). The relative mRNA levels of the target genes are presented as previously described [34].

### Migration assay

A total of 3*10^5^ VSMCs in 6 cm dish were treated with vehicle or PM_2.5_ with and without inhibitors in 3 ml of 0.5% FBS medium for 48 h. A total of 3*10^4^ cells were then re-seeded in the upper chamber of 24-well Transwell cell culture plates (Millipore, Billerica, MA, USA; pore size, 8 μm) with 0.5 ml 10% FBS medium. The bottom chambers were filled with 1 ml media containing platelet-derived growth factor (PDGF)-BB (Peprotech, Rocky Hill, NJ, USA; 10 ng/mL) as a chemoattractant. After incubation for 4 h, the upper layer of the cells was scraped off using sterile cotton swabs, and the cells in the lower layer of the membrane were fixed and stained with 0.1% crystal violet (Sigma-Aldrich, St. Louis, MO, USA). Cells that migrated to the underside of the membrane were visualized under a microscope. Image analyses were performed using MetaMorph 7.8.11.0 software (Molecular Devices, San Jose, CA, USA).

### Enzyme-linked immunosorbent assay

OPN and IL-6 concentration in the media and BALF were measured using enzyme-linked immunosorbent assay (ELISA) kits for mice (R&D Systems, Minneapolis, MN, USA) in accordance with the manufacturer’s instructions.

### NF-κB reporter gene assay

A total of 5*10^4^ VSMCs in 12 well were treated with vehicle or PM_2.5_ with or without inhibitors in 0.5% FBS media for 48 h. For the luciferase assays, VSMCs were transfected with pNF-κB-Luc and pCMV-β-gal using Lipofectamine 2000 (Invitrogen, Foster City, CA, USA), according to the manufacturer’s protocol. The transcriptional activity was determined using the Luciferase Assay System (Promega, Madison, WI, USA) and a luminometer (Berthold Analytical Instruments, Nashua, NH, USA).

### Calculation of the contribution of each PAH in PM_2.5_ to AhR activation

To quantitatively assess the contribution of each ambient PM_2.5_-bound PAH to AhR agonist activity, the toxic equivalent (TEQ) approach was applied. TEQs express the toxicity of a chemical mixture in terms of an equivalent concentration of a reference chemical, with the assumption that chemicals exert toxicity through the same biological or toxic pathway and that the effects of mixtures are additive [35].

In this study, we calculated the TEQs to BaP (expressed as BaP-TEQs) to assess the potential effects of PAH mixtures on AhR activation by integrating PM_2.5_-bound PAH concentrations (C) and relative potencies (REP) of individual PAHs in the AhR pathway. Additional file 1: Table 1 presents the ambient concentrations of seven PAHs in PM_2.5_ from January to March 2018. The AC_50_ values (Additional file 1: Table 2) obtained from the U.S. EPA’s ToxCast database were used to calculate the REP_*i*_ by dividing the AC_50_ of BaP (the reference compound) by the AC_50_ of other PAHs. In addition, REP_*i*_ values determined based on EC_50_ concentrations in reporter gene assays employing human hepatoma HepG2-AZ-AhR cells by Vondráček et al. were also used to calculate BaP-TEQs [36]. The formula for calculating the BaP-TEQs (ng/m^3^) of the seven PAHs in PM_2.5_ is as follows:

1 $${\text{BaP}} - {\text{TEQs}} = \mathop \sum \limits_{i}^{n} \left( {{\text{C}}_{i} \times {\text{REP}}_{i} } \right)$$

where C_*i*_ is the measured concentration of airborne PM-bound PAH compound *i* (ng/m^3^) and REP_*i*_ is the AhR-inducing relative potency of PAH compound *i*. The Monte Carlo simulation with 10,000 iterations was implemented using Oracle Crystal Ball software (version 11.1, Oracle Corporation, Redwood Shores, CA, USA) to quantify the uncertainty of BaP-TEQs through the random sampling method from the probability distribution of C_*i*_. Lognormal distribution was assumed for C_*i*_. The relative percent contribution of each PAH to BaP-TEQs was then calculated to identify which PAH contributed the most to AhR activation.

### Human participants

The study comprised 72 healthy participants aged 20–35 years from Neihu and Shijuang districts in the Taipei metropolitan area. We excluded seven participants with a smoking history and eight without a complete PM_2.5_ assessment and ELISA analysis. Finally, 57 participants who were nonsmokers and free of cardiopulmonary diseases were included in the subsequent analysis. The study protocol was approved by the Institutional Review Board of the National Health Research Institutes, Taiwan (NIRB File Number: EC1020205). The constitution and operation of review board are formulated according to the guidelines of the ICH-GCP. After written informed consent was obtained from individual participants, each participant wore a personal PM_2.5_ sampler to assess the PM_2.5_ concentration in the breathing zone over a 24-h period (from 8:00 AM on day 1 to 8:00 AM on day 2). On the following morning (8:00 AM to 10:00 AM on day 2), blood and urine samples of the participants were collected, and they completed a self-administered questionnaire regarding demographic information, lifestyle habits, and previous and current diseases.

### PM_2.5_ personal exposure assessment

Personal air sampling was performed by a 2.5-µm impactor (PEM; SKC Inc., PA, USA) and a pump (Gilian Gilair, Sensidyne Inc., FL, USA). We collected personal air samples using a quartz fibre filter (2500 QAT-UP, Purtram, Conn., USA). After the air was passed through a electrostatic neutralizer, the filters were weighed. We used a microbalance (Mettler-Toledo, MT5, Greifensee, Switzerland) with a reading of 1-µg to analyze the weight. The relative humidity in the laboratory was 60% and the temperature was constant. The detection limit for mass concentration was 2.11 µg/m^−3^.

### Plasma OPN measurements

A 10-mL fasting blood sample was collected on the morning of the visiting day. Subjects were asked to fast at least 8 h before the blood sample is taken. These blood samples were centrifuged at 1700*xg* for 15 min and then stored at − 80 °C before assay. We measured plasma OPN levels using the Solid Phase Sandwich ELISA (Catalog No.: DOST00-R&D Systems). The assays were performed in a 96-well microplate spectrophotometer SPECTRAMax 190 (Molecular Devices, Sunnyvale, CA, USA).

### Urinary 1-hydroxypyrene measurements

A 50-mL urine sample was collected from the participants in a Falcon 50-mL polypropylene conical tube (Corning Science, Reynosa, Tamaulipas, Mexico) on the morning of the visiting day. The samples were stored in a cooler with freezer blocks and maintained at − 80 °C until further analysis. After the samples were thawed and centrifuged, urinary 1-hydroxy pyrene (1-OHP) was separated through high-performance liquid chromatography by using a method developed by Jongeneelen and colleagues [37–39]. The detection limit in this method was 5.43 ng based on seven repeated measurements at a concentration of 15.0 ng/dL. The coefficient of variation in this method ranged from 1.87% to 8.40%.

### Statistical analysis

Statistical analyses were performed using SPSS 24.0 software (SPSS, Chicago, IL, USA). The treatment and control groups were compared using one-way or two-way analysis of variance followed by Tukey’s range test in SPSS Statistics (significance: *p* < 0.05). In the human study, the plasma OPN and 1-OHP concentrations exhibited left-skewed distributions; therefore, the natural logarithm of the original data was used to transform the data to normal distribution. We used one-way ANOVA and P trend analysis to assess the difference and trend for means. Fisher’s least significant difference (LSD) method was used in post hoc comparisons to test the influence of different 1-OHP groups on OPN levels. The linear regression model was used to analyze the relationship between 1-OHP levels and OPN levels after adjustment for covariates.

## Results

### AhR activation involved in PM_2.5_-induced VSMC migration and OPN expression

**Fig. 1:**
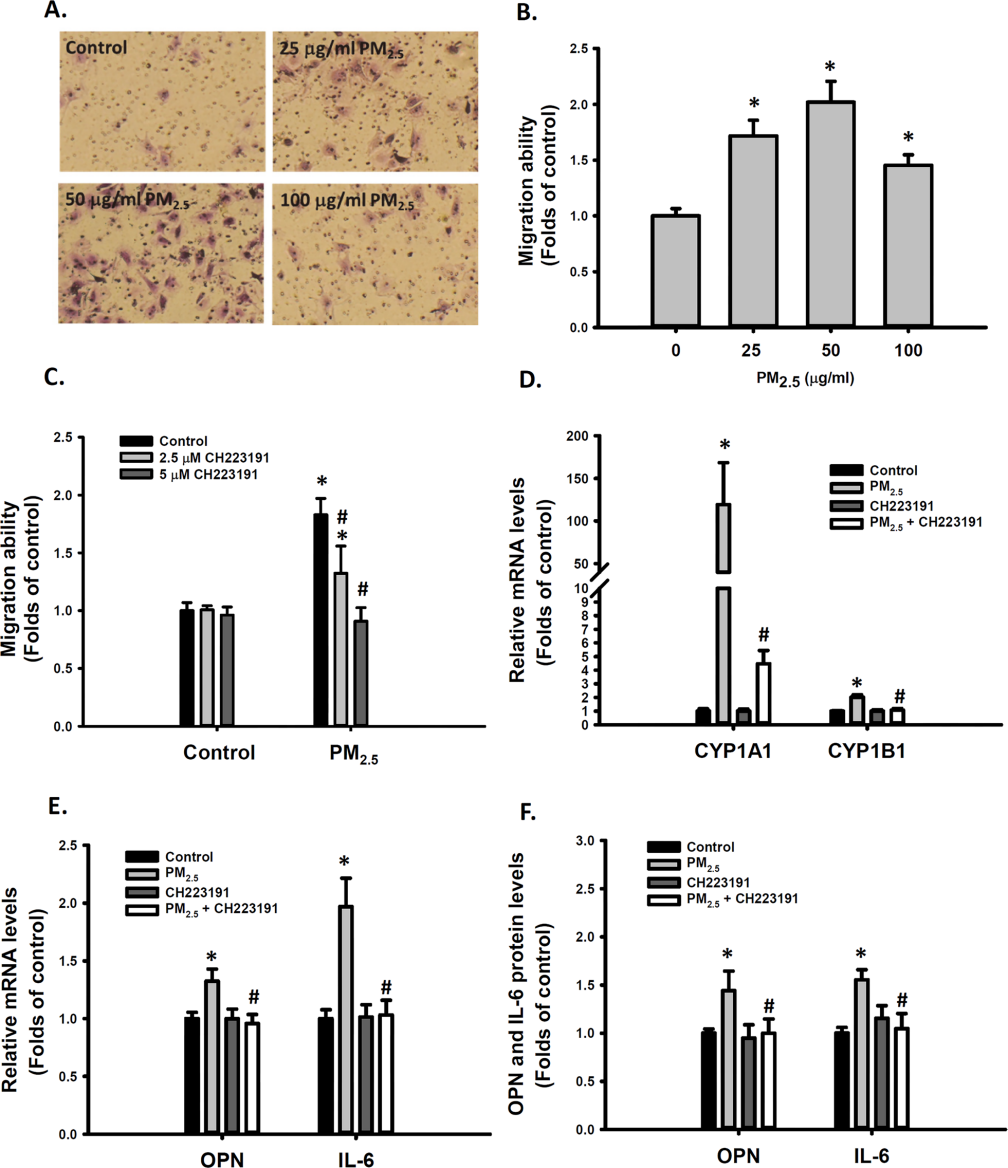
Involvement of AhR activation in PM_2.5_-induced cell migration ability and OPN and IL-6 expression in VSMCs. VSMCs were treated with d_2_H_2_O or 25, 50, and 100 μg/mL PM_2.5_ for 48 h. Migration abilities of VSMCs were quantified (**A**, **B**). VSMCs were treated with d_2_H_2_O or 25 μg/mL PM_2.5_ with or without 2.5 or 5 μM CH223191 for 48 h. Migration abilities of VSMCs were measured and quantified and are shown in (**C**). VSMCs were treated with d_2_H_2_O or 25 μg/mL PM_2.5_ with and without 5 μM CH223191 for 48 h. The following parameters were determined: **D** *CYP1A1* and *CYP1B1* relative mRNA levels; **E** *OPN* and *IL-6* relative mRNA levels; and **F** OPN and IL-6 protein levels in the media. The results are presented as mean ± standard deviation (SD) for three independent experiments with 2–4 replicates in each experiment. *p < 0.05 compared with the d_2_H_2_O-treated cells (n = 3, one-way ANOVA). #*p* < 0.05 compared with the PM_2.5_-treated cells (n = 3, two-way ANOVA). Control: d_2_H_2_O treatment as control

Our previous animal studies demonstrated that PM_2.5_ induced medial thickening and intimal hyperplasia of these small arteries in mice lung, suggesting PM_2.5_ induced VSMC migration [7, 9]. We previously also demonstrated that exposure to ambient PM_2.5_ increased the migration ability of VSMCs [7] and activated the AhR pathway in vitro [40]*.* In the present study, we investigated whether AhR activation plays a role in PM_2.5_-induced VSMC migration. First, exposure to 25–100 μg/mL PM_2.5_ significantly increased the migration ability of VSMCs (Fig. 1A, B). Cotreatment with 2.5 or 5 μM CH223191, an AhR antagonist, prevented the increase in the migratory ability of VSMCs induced by 25 μg/mL PM_2.5_ (Fig. 1C). Typically, *CYP1A1* and *CYP1B1* genes were transcriptionally regulated by AhR activation [21]. Furthermore, PM_2.5_ significantly increased *CYP1A1* and *CYP1B1* mRNA levels in VSMCs, and this increase was prevented by cotreatment with 5 μM CH223191 (Fig. 1D). Some studies have indicated that PM induces inflammatory responses and increases IL-6 levels in macrophages [41] and vascular endothelial cells [42]. We previously reported that exposure to ambient PM_2.5_ increased OPN and IL-6 expression in VSMCs as well as in mice (Ho et al., 2019). Cotreatment with CH223191 significantly reduced 25 μg/mL PM_2.5_-induced increase in OPN and IL-6 mRNA levels in VSMCs (Fig. 1E). Similarly, PM_2.5_-induced increase in OPN and IL-6 protein levels was inhibited by CH223191 in VSMCs (Fig. 1F). As a control experiment, the extract of blank fiberglass filter didn’t cause cytotoxicity of VSMCs or modulate gene expression in VSMCs, including OPN, IL-6 and CYP1B1 (Additional file 1: Figure S4). These results suggest that AhR activation is involved in the mechanism of PM_2.5_-enhanced VSMC migration ability and OPN and IL-6 expression.

### Cross-talk of the AhR and NF-κB pathways in PM2.5-treated VSMCs

**Fig. 2:**
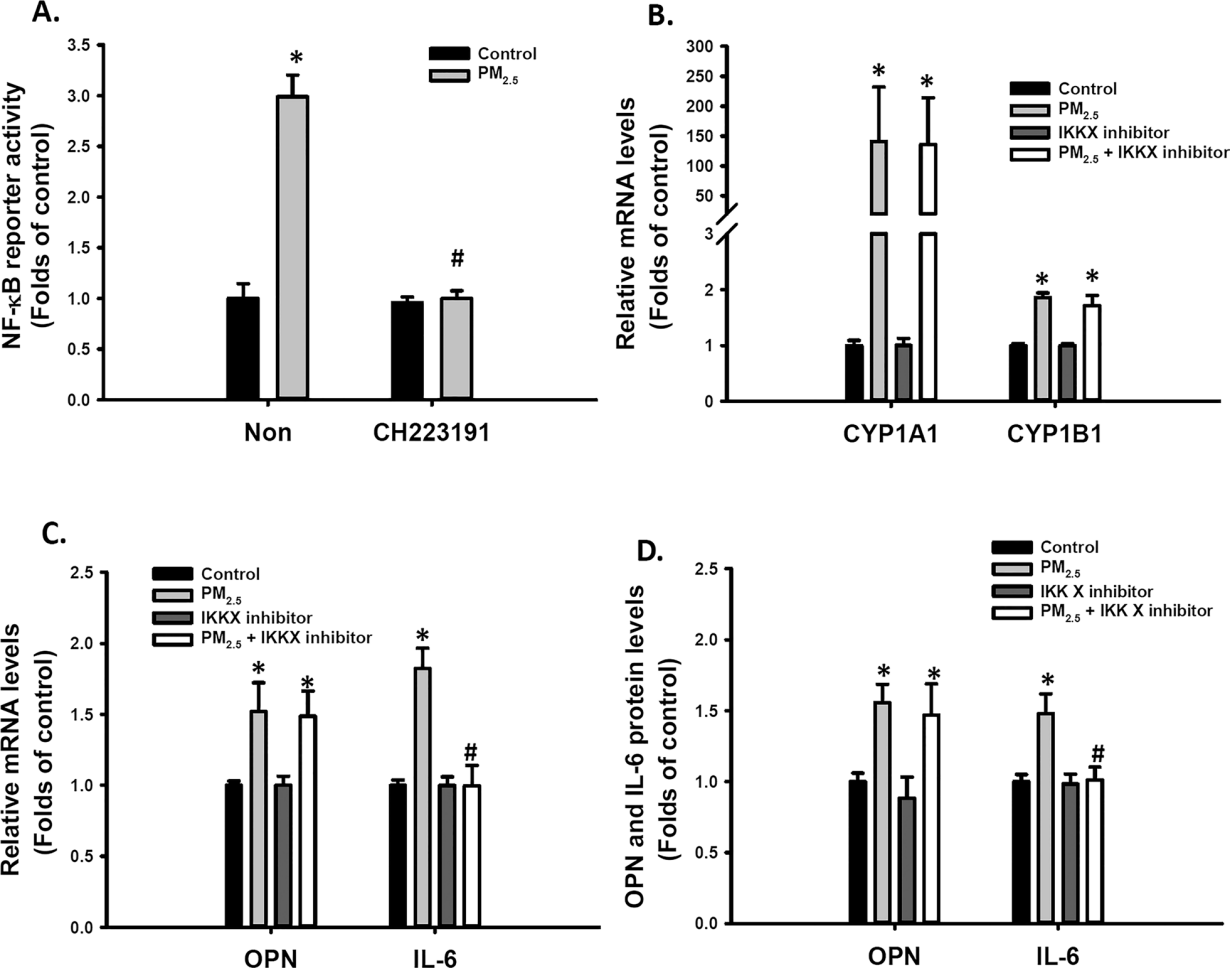
Cross-talk between the AhR and NF-κB pathways in PM_2.5_-treated VSMCs. VSMCs were treated with d_2_H_2_O or 25 μg/mL PM_2.5_ with and without 5 μM CH223191 for 48 h. **A** The NF-κB reporter activity was measured. VSMCs were treated with d_2_H_2_O or 25 μg/mL PM_2.5_ with and without 10 μM IKK inhibitor for 48 h. The following parameters were determined: **B** *CYP1A1* and *CYP1B1* relative mRNA levels; **C** *OPN* and *IL-6* relative mRNA levels; and **D** OPN and IL-6 protein levels in the media. The results are presented as mean ± SD for three independent experiments with 2–4 replicates in each experiment.*p < 0.05 compared with the d_2_H_2_O-treated cells(n = 3, one-way ANOVA). #*p* < 0.05 compared with the CH223191 or IKK inhibitor-treated cells (n = 3, two-way ANOVA). Non: no treatment with CH223191. Control: d_2_H_2_O treatment as control

We previously reported that PM_2.5_ increased the migration ability of VSMCs through the NF-κB dependent pathway [7]. We investigated whether a cross-talk between the PM_2.5_-activated AhR and NF-κB pathways occurred in VSMCs. Cotreatment with 5 μM CH223191 for 48 h completely blocked the 25 μg/mL PM_2.5_-induced increase in NF-κB activity (Fig. 2A). However, 10 μM IKK inhibitor, an inhibitor of NF-κB activation, failed to prevent PM_2.5_-induced high *CYP1A1* and *CYP1B1* expression (Fig. 2B). Although cotreatment with 10 μM IKK inhibitor prevented the PM_2.5_-induced increase in IL-6 mRNA and protein levels (Fig. 2C, D)*,* it failed to prevent the PM_2.5_-induced increase in OPN relative to mRNA and protein levels (Fig. 2C, D). These results suggest that AhR is involved in the NF-κB pathway activation mechanism by PM_2.5_. The NF-κB pathway involved PM_2.5_-induced IL-6 secretion, but not the OPN secretion levels induced by PM_2.5_.

### Organic PM_2.5_ extract increased cell migration ability and OPN expression in VSMCs

**Fig. 3:**
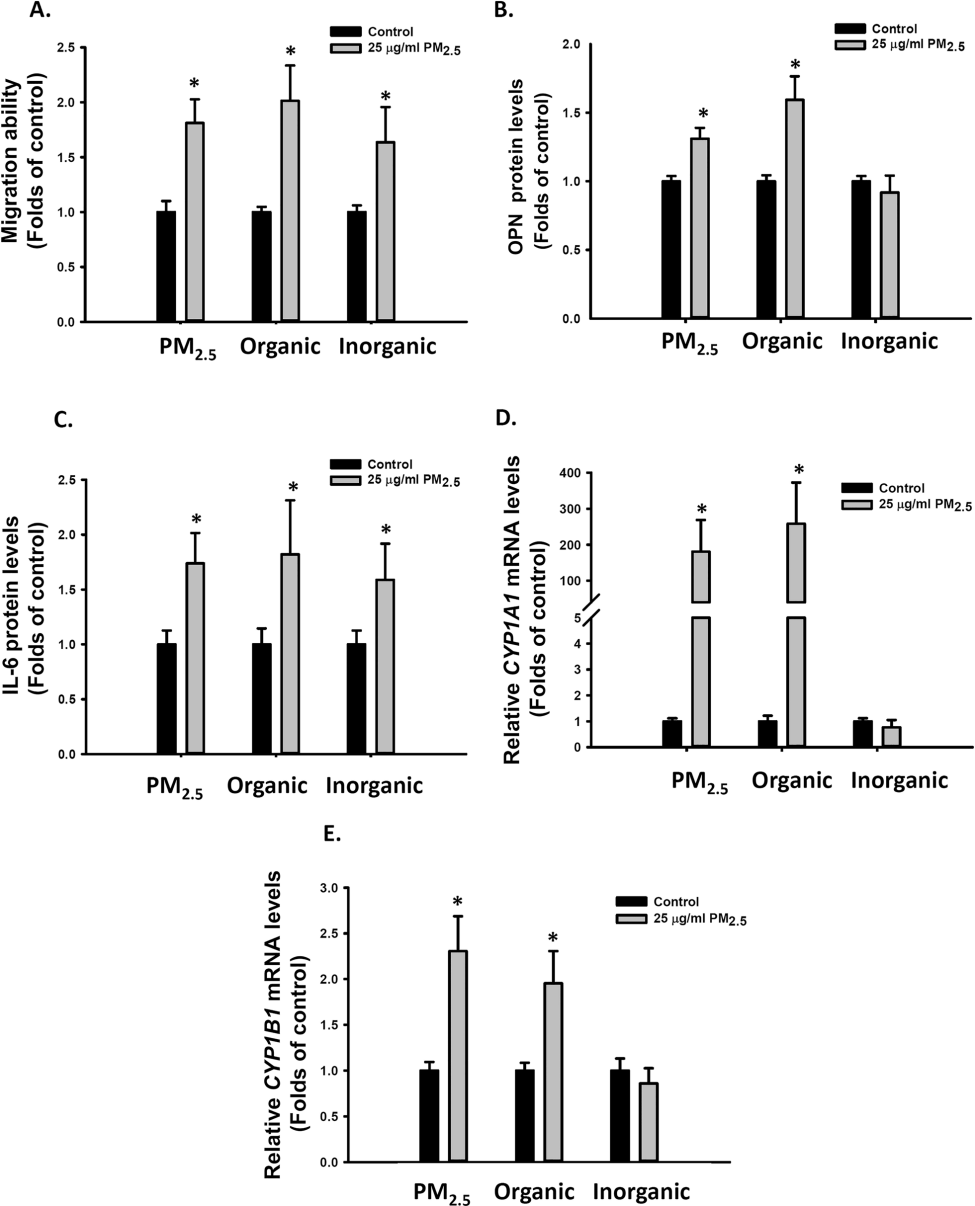
Effects of the organic and inorganic extracts of PM_2.5_ on cell migration ability and OPN and IL-6 expression in VSMCs. VSMCs were treated with d_2_H_2_O, DMSO, 25 μg/mL PM_2.5_, and organic or inorganic extract of PM_2.5_ for 48 h. The following parameters were determined: **A** migration abilities of VSMCs; **B** OPN protein levels in the media; **C** IL-6 protein levels in the media; **D** relative *CYP1A1* mRNA levels; and **E** relative *CYP1B1* mRNA levels. The results are presented as mean ± SD for three independent experiments with 2–4 replicates in each experiment. *p < 0.05 compared with the d_2_H_2_O- or DMSO-treated cells(n = 3, one-way ANOVA). Control: d_2_H_2_O or DMSO treatment as control

PM_2.5_ is a complex mixture. To determine which PM_2.5_ components increased the migration ability of VSMCs, we extracted the organic (containing PAHs) and inorganic (metals) components from PM_2.5_ (Additional file 1: Tables 3 and 4). A total of 22 PAHs were quantified, of which approximately 88% were extracted from the organic extract of PM_2.5_ with hexane/acetone (Additional file 1: Table 3). Next, the VSMCs were treated with organic or inorganic extracts at a dose equivalent to 25 μg/mL PM_2.5_. Similar to intact PM_2.5_, the organic extract of PM_2.5_ also enhanced the migratory ability of VSMCs (Fig. 3A), increased OPN mRNA and protein levels (Additional file 1: Figure S1A, S2A and 3B), increased IL-6 mRNA and protein levels (Additional file 1: Figure S1B, S2B and 3C), and increased *CYP1A1* and *CYP1B1* relative mRNA levels in VSMCs (Additional file 1: Figure S2C, S2D, 3D and 3E). By contrast, the inorganic extract of PM_2.5_ enhanced the migratory ability of VSMCs (Fig. 3A) and increased *IL-6* mRNA and protein levels (Additional file 1: Figure S1B, S2B and 3C) but failed to increase *OPN*, *CYP1A1,* or *CYP1B1* relative mRNA levels in VSMCs (Additional file 1: Figure S1A,S2A,S2C-D, 3D and 3E). Thus, both the organic and inorganic components of PM_2.5_ increased IL-6 expression. However, the organic components of PM_2.5_ also contributed to OPN induction and considerably increased VSMC migration ability.

### CYP1B1 is involved in PM_2.5_-induced VSMC migration and OPN expression

**Fig. 4:**
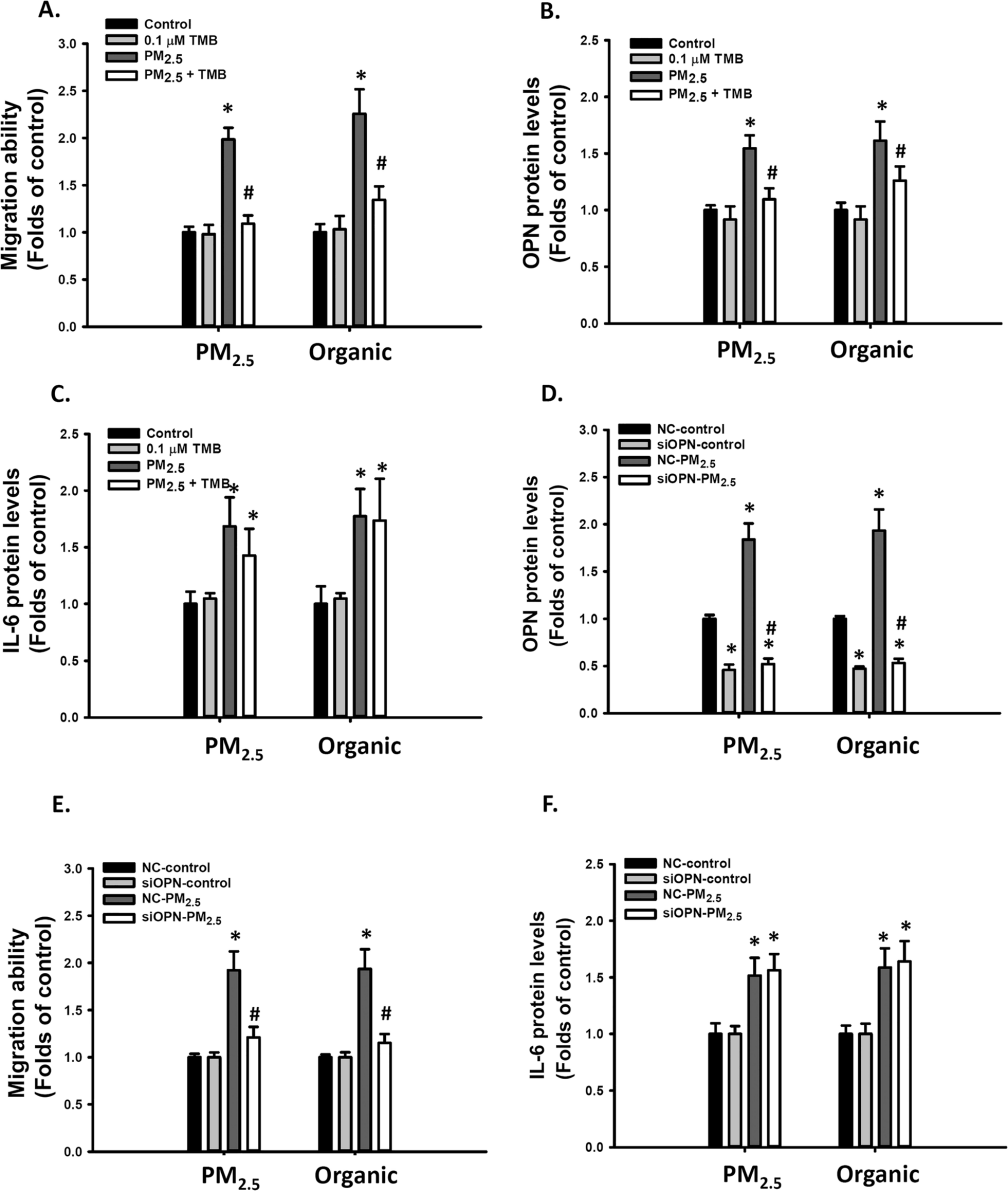
PM_2.5_ and its inorganic extract induced cell migration ability through CYP1B1 and OPN in VSMCs. VSMCs were treated with d_2_H_2_O, DMSO, 25 μg/mL PM_2.5_, and organic extract of PM_2.5_ with or without 0.1 μM TMB for 48 h. The following parameters were determined: **A** migration abilities of VSMCs; **B** OPN protein levels in the media; and **C** IL-6 protein levels in the media. The results are presented as mean ± SD for three independent experiments with 2–4 replicates in each experiment. *p < 0.05 compared with the Control-treated cells (n = 3, one-way ANOVA). #*p* < 0.05 compared with the CYP1B1 inhibitor-treated cells (n = 3, two-way ANOVA). Control: d_2_H_2_O or DMSO treatment as control. NC-VSMCs and siOPN-VSMCs were treated with d_2_H_2_O, DMSO, 25 μg/mL PM_2.5_, and organic extract of PM_2.5_ for 48 h. The following parameters were determined: **D** migration abilities of VSMCs; **E** OPN protein levels in the media; and **F** IL-6 protein levels in the media. The results are presented as mean ± SD for three independent experiments with 2–4 replicates in each experiment.*p < 0.05 compared with the NC-treated cells(n = 3, one-way ANOVA). #*p* < 0.05 compared with NC PM_2.5_-treated cells (n = 3, two-way ANOVA). NC: negative control, siOPN: OPN siRNA, Control: d_2_H_2_O or DMSO treatment as control

CYP1B1 is upregulated by AhR activation and is critical for neointimal growth in vascular injury [43]. We investigated whether it plays a role in PM_2.5_-induced VSMC migration and OPN expression. Cotreatment with 0.1 μM TMB, a CYP1B1 inhibitor, prevented the increase in migratory ability of VSMCs (Fig. 4A) and OPN mRNA and protein levels induced by PM_2.5_ and its organic extract (Additional file 1: Figure S3A and 4B) but failed to prevent the PM_2.5_-induced increase in *IL-6* relative mRNA and protein levels (Additional file 1: Figure S3B and 4C) in VSMCs. These results suggest that PM_2.5_-induced AhR activation might increase VSMC migration ability and OPN expression through the CYP1B1-dependent mechanism.

### Inhibiting OPN induction prevented PM_2.5_-induced increase in VSMC migration

To understand whether OPN plays a role in PM_2.5_-induced VSMC migration, we knocked down OPN expression through siRNA. siRNA partially reduced the mRNA and protein levels of OPN to 30% and 55% of those of controls, respectively (Additional file 1: Figure S3C and 4D). OPN levels induced by PM_2.5_ and its organic extract were drastically reduced to 30% and 40%, respectively, at the mRNA level (Additional file 1: Figure S3C) and 38% and 47%, respectively, at the protein level (Fig. 4D). Similarly, OPN knockdown reduced the migratory ability induced by PM_2.5_ and its organic extract to 37% and 39%, respectively, of those of controls (Fig. 4E), but it failed to prevent the PM_2.5_-induced increase in *IL-6* mRNA and protein levels (Additional file 1: Figure S3D and 4F) in VSMCs. These results suggest that OPN contributed to PM_2.5_-induced enhancement of VSMC migration ability.

### PM_2.5_-bound PAHs increased OPN secretion and VSMC migration ability

**Fig. 5:**
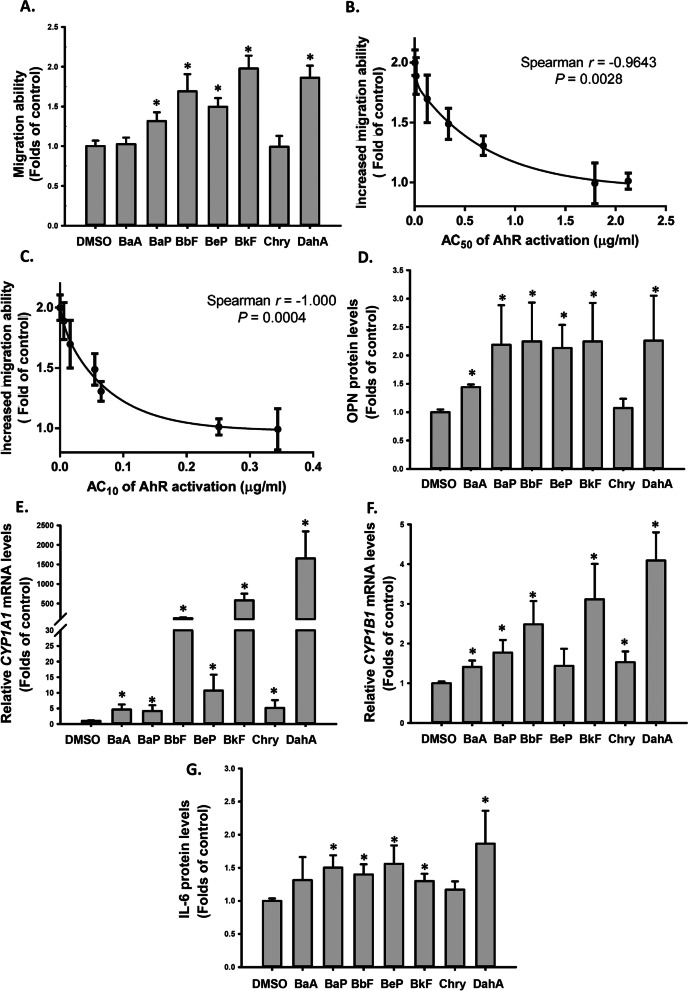
PM_2.5_-bound PAHs induced cell migration and OPN and IL-6 expression in VSMCs. VSMCs were treated with DMSO and 0.3μg/mL BaA, BaP, BbF, BeP, BkF, chrysene, or DahA for 48 h. The following parameters were determined: **A** migration abilities of VSMCs; **B** correlation between the AC_50_ of AhR activation in the ToxCast and PM_2.5_-induced migration abilities in VSMCs; **C** correlation between the AC_10_ of AhR activation in the ToxCast and PM_2.5_-induced migration abilities in VSMCs; **D** OPN protein levels in the media; **E** *CYP1A1* relative mRNA levels; **F** *CYP1B1* relative mRNA levels; and **G** IL-6 protein levels in the media. The results are presented as mean ± SD for three independent experiments with 2–4 replicates in each experiment.*p < 0.05 compared with the DMSO-treated cells (n = 3, one-way ANOVA). Control: DMSO treatment as control

PAHs in PM_2.5_ are major AhR agonists. We further investigated whether AhR activation potencies of PM_2.5_-bound PAHs were correlated with their abilities to enhance the migration ability of VSMCs. We searched for the AhR reporter activities of 22 PAHs in the ToxCast HTS database. Eight out of 22 PAHs were indicated to be active in the AhR activity assay in the ToxCast database. However, the AC_50_ of AhR activity for acenaphthylene (91.5 μM) was considerably higher than AC_50_ of the other seven PAHs (0.05–9.31 μM). The seven PAHs with AhR agonistic activity are benz(a)anthracene (BaA), benzo(a)pyrene (BaP), benzo(b)fluoranthene (BbF), benzo(e)pyrene (BeP), benzo(k)fluoranthene (BkF), chrysene, and dibenz(a,h)anthracene (D(a,h)A) (Additional file 1: Table 2). When VSMCs were treated with 0.3 μg/mL of the seven PAHs individually, the migration ability of the VSMCs was significantly increased (Fig. 5A), which was well correlated with the AC_50_ and AC_10_ of AhR activation by these PAHs reported in the ToxCast database (Figs. 5B and 4C). Furthermore, the seven PAHs increased OPN and IL-6 protein secretion and *CYP1A1* and *CYP1B1* mRNA levels in the VSMCs (Fig. 5D–G). These results supported our hypothesis that some PM_2.5_-bound PAHs enhanced VSMC migration through the AhR activation-dependent pathway.

### Contribution of individual PM_2.5_-bound PAHs to the AhR activation

On the basis of the AC_50_ of AhR activation by PAHs reported in the ToxCast database (Additional file 1: Table 2), the potencies of the effective AhR agonists (i.e., REP) for the seven PAHs were calculated, and the order of REPs was BkF > D(a,h)A > BbF > BeP > BaP > CHR ~ BaA (Additional file 1: Table 2). The rank-order of REPs derived from the AZ-AhR assay was largely similar to that derived from the AhR-CALUX assay in the ToxCast, indicating that BkF, D(a,h)A, and BbF were the most potent PAHs (Table 1).

**Table 1:** Ambient concentrations of selected PAHs in PM_2.5_ and their BaP-TEQs and contributions of PAHs to BaP-TEQs based on relative potencies (REPs) determined in AhR-CALUX and AZ-AhR assays

Compound	Abbreviation	PAH concentrations in PM_2.5_ (ng/m^3^)	ToxCast human HepG2-based AhR-CALUX cells			Human HepG2-AZ-AhR cells		
			REP^a^	BaP-TEQs (ng/m^3^)^b^	Contribution of PAHs to total BaP-TEQ (%)	REP^c^	BaP-TEQs (ng/m^3^)^b^	Contribution of PAHs to total BaP-TEQ (%)
Benz(a)anthracene	BaA	0.07 ± 0.02	0.29	0.02 ± 0.01	0.08 ± 0.03	0.60	0.04 ± 0.01	0.63 ± 0.19
Chrysene	CHR	0.19 ± 0.04	0.34	0.07 ± 0.01	0.24 ± 0.08	1.50	0.29 ± 0.06	4.09 ± 1.21
Benzo(b)fluoranthene	BbF	0.30 ± 0.08	5.44	1.63 ± 0.42	5.83 ± 2.00	4.00	1.19 ± 0.32	16.61 ± 4.85
Benzo(k)fluoranthene	BkF	0.12 ± 0.03	213.39	25.81 ± 7.41	86.52 ± 3.87	43.20	5.23 ± 1.52	69.61 ± 6.87
Benz(*e*)pyrene	BeP	0.22 ± 0.06	2.02	0.44 ± 0.13	1.57 ± 0.60	NA	–	–
Benzo(*a*)pyrene	BaP	0.13 ± 0.03	1.00	0.13 ± 0.03	0.47 ± 0.17	1.00	0.13 ± 0.03	1.85 ± 0.59
Dibenz(*a,h*)anthracene	D(a,h)A	0.03 ± 0.01	49.45	1.49 ± 0.51	5.29 ± 2.16	17.20	0.52 ± 0.18	7.22 ± 2.71
Total		1.07 ± 0.25	–	29.59 ± 7.45	100	–	7.41 ± 1.57	100

Values are presented as mean ± standard deviation. *NA* not available

^a^Calculated by dividing AC_50_ of BaP (the reference compound) by the AC_50_ of other PAHs

ToxCast’s AC_50_ were provided in the Additional file 1: Table 2

^b^Calculated using Eq. (1)

^c^Adopted from Vondráček et al. (2017)

However, the REP values of BkF and D(a,h)A determined using the AhR-CALUX assay were 213.39 and 49.45, respectively, which were higher than those determined using the AZ-AhR assay (43.20 and 17.20, respectively). As a result, the calculated total BaP-TEQs of the PAH mixtures in PM_2.5_ based on the AhR-CALUX assay-derived REPs (mean ± standard deviation: 29.59 ± 7.45 ng/m^3^) were higher than those based on the AZ-AhR assay-derived REPs (7.41 ± 1.57 ng/m^3^) (Table 1). Nevertheless, the relative contribution of individual PAH to total BaP-TEQs calculated using both the AhR-CALUX and AZ-AhR assays demonstrated that BkF (AhR-CALUX: 86.52 ± 3.87%; AZ-AhR: 69.61 ± 6.87%) was the most important contributor to the AhR activation, followed by BbF (5.83 ± 2.00%; 16.61 ± 4.85%) and D(a,h)A (5.29 ± 2.16%; 7.22 ± 2.71%) (Table 1). Overall, these compounds (BkF + BbF + D(a,h)A) together accounted for more than 93% of the total BaP-TEQs, of which BkF + BbF contributed to more than 86%.

### PM_2.5_-induced pulmonary inflammation and OPN secretion was alleviated in mice with low AhR affinity

**Fig. 6:**
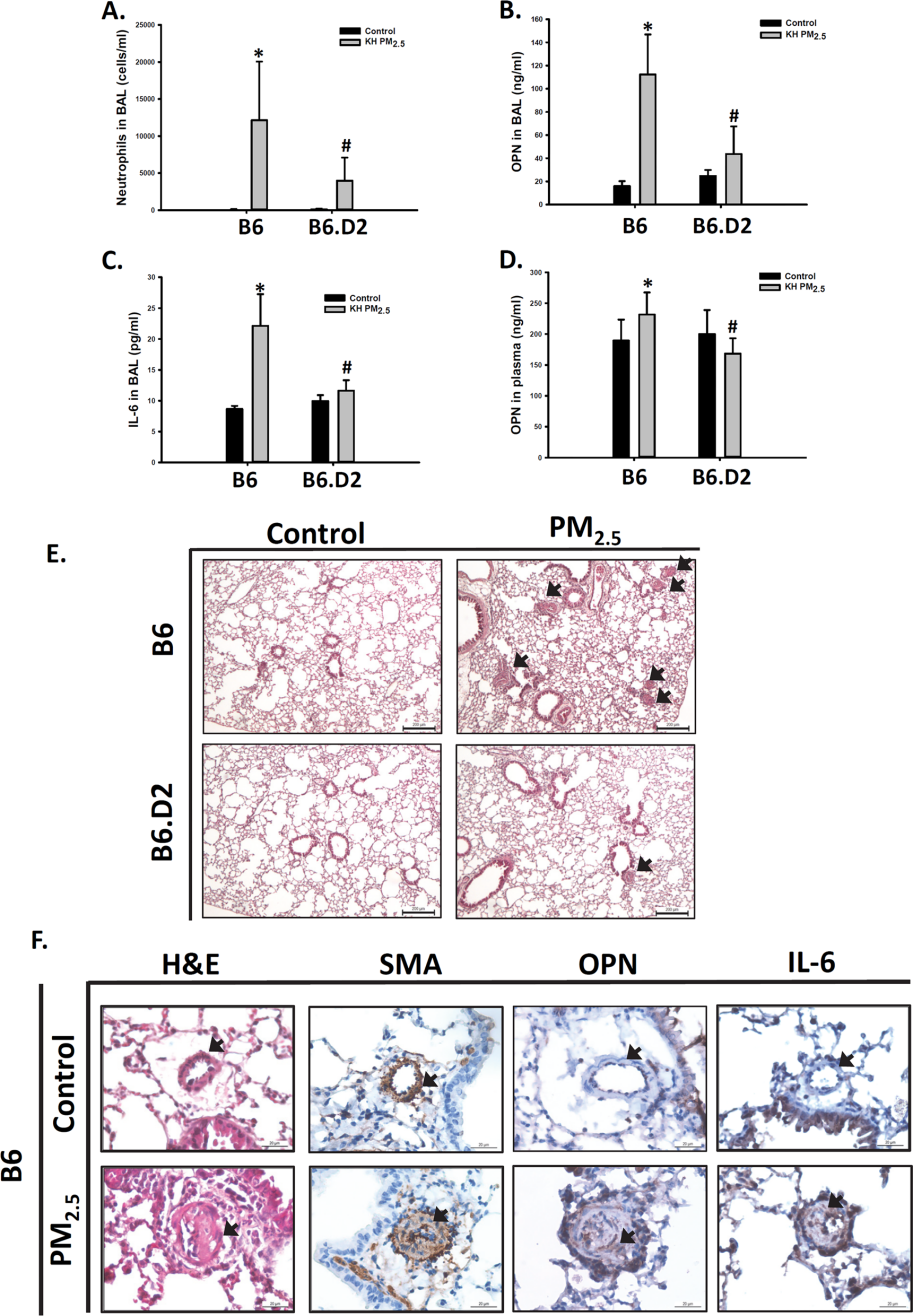
PM_2.5_-induced vascular remodeling, pulmonary inflammation, and IL-6 and OPN secretion was reduced in mice with low AhR affinity. The B6 and B6.D2 mice were aspirated with 25 μg of PM_2.5_ twice weekly for 12 weeks. **A** neutrophil numbers, **B** OPN protein in BALF, **C** IL-6 protein in BALF, and **D** OPN protein concentrations in plasma were determined. **E** Lung sections were stained with H&E. The arrow indicates the lesions of vascular remodeling. Scale bar, 200 μm. **F** The B6 mice was aspirated with 25 μg of PM_2.5_ twice weekly for 12 weeks. H&E; SMA staining; IHC staining of OPN; and IHC staining of IL-6. Arrows indicate small arteries. Scale bar, 30 μm. Each value represents the mean ± SD of ten mice. *P < 0.05 for comparison with the control-treated mice (n = 10, one-way ANOVA); ^**#**^*P* < 0.05 for comparison between B6 and B6.D2 mice (n = 10, two-way ANOVA)

Previously we reported that oropharyngeal aspiration of 25 μg PM_2.5_ significantly causes medial thickening and intimal hyperplasia in small pulmonary arteries in mice [7, 9]. Therefore, we selected 25 μg as the dose to investigate the effect of PM_2.5_ on AhR pathway activation in this study. We performed a preliminary study that mice were administrated with double distilled water as vehicle control, blank filter extract as filter control or 25 μg PM_2.5_ per mouse twice per week for 8 weeks. We observed that filter control did not change the total cell numbers or the OPN level in BALF, but PM_2.5_ significantly increased the total cell numbers and the OPN level in BALF, as compared with vehicle or filter control (Additional file 1: Figure S4). We further compared the effects of PM_2.5_ in mice with different allelic variants of the AhR. B6.D2NAhrd/J (B6.D2) that carry the Ahr^d^ allele originating from the DBA2/N donor strain on a C57BL/6 inbred background. The Ahr^d^ allele expresses the low-affinity ligand-binding form of the AhR [44]. B6 and B6.D2 mice were treated with PM_2.5_ for 12 weeks. The respiratory volume of B6 mice for 7 days reaches a total of 0.375 m^3^ [45]. Exposure of 25 μg/mouse twice per week was approximately equivalent to 125 μg/ m^3^/day in this study. Neutrophil numbers in the BALF were tremendously elevated in B6 mice. The increase in neutrophil numbers was considerably lower in B6.D2 mice than in B6 mice (Fig. 6A). Similarly, PM_2.5_ exposure significantly increased OPN and IL-6 protein secretion in the BALF of B6 mice, and the increases were considerably lower in B6.D2 mice than in B6 mice (Fig. 6B, C). PM_2.5_ exposure also significantly increased OPN protein concentrations in the plasma of B6 mice, and the increases were considerably lower in B6.D2 mice than in B6 mice (Fig. 6D). However, IL-6 protein was undetectable in the plasma of both mice. The incidence of medial thickening and intimal hyperplasia in small pulmonary arteries was also lower in B6.D2 mice (Fig. 6E). In addition, PM_2.5_ also induced OPN and IL-6 protein in remodeled small pulmonary arteries of B6 mice (Fig. 6F).

### Plasma OPN concentrations were associated with urinary 1-OHP concentration in a human population

We previously reported that exposure to PM_2.5_ was positively correlated with plasma OPN levels in young adults [9]. In the present study, we monitored the urinary PAH metabolite (1-OHP) and measured plasma OPN concentrations in 57 nonsmoking young adults recruited from the metropolitan area. The characteristics of the study participants are presented in Table 2. More than half of the participants were women (56.1%), and 87.7% and 77.2% of the participants did not have a regular habit of alcohol consumption and vitamin supplement intake, respectively. The participants’ average age and body mass index (BMI) were 24.2 years and 23.0, respectively. The participants’ average time spent indoors, time spent outdoors, and transit time were 19.5 (81.1%), 3.82 (16.9%), and 0.70 (3%) h, respectively.

**Table 2:** Characteristics of non-smoking study population (n = 57)

Variables	Study subjects	
	Mean	SD
Age (year)	34.4	3.2
BMI (Kg/m^2^)	23.0	3.6
Waist circumstances (cm)	78.4	9.9
Time spent indoors (hours)	19.5	3.6
Time spent outdoors (hours)	3.82	2.65
Transit time (hours)	0.70	0.66

	N	%
Gender		
Female	32	56.1
Male	25	43.9
Alcohol drinking		
No	50	87.7
Yes	7	12.3
Vitamin supplement		
No	44	77.2
Yes	13	22.8
Medicine taken		
No	54	94.7
Yes	3	5.3
Regular exercise		
No	2	3.5
Yes	55	96.5

**Fig. 7:**
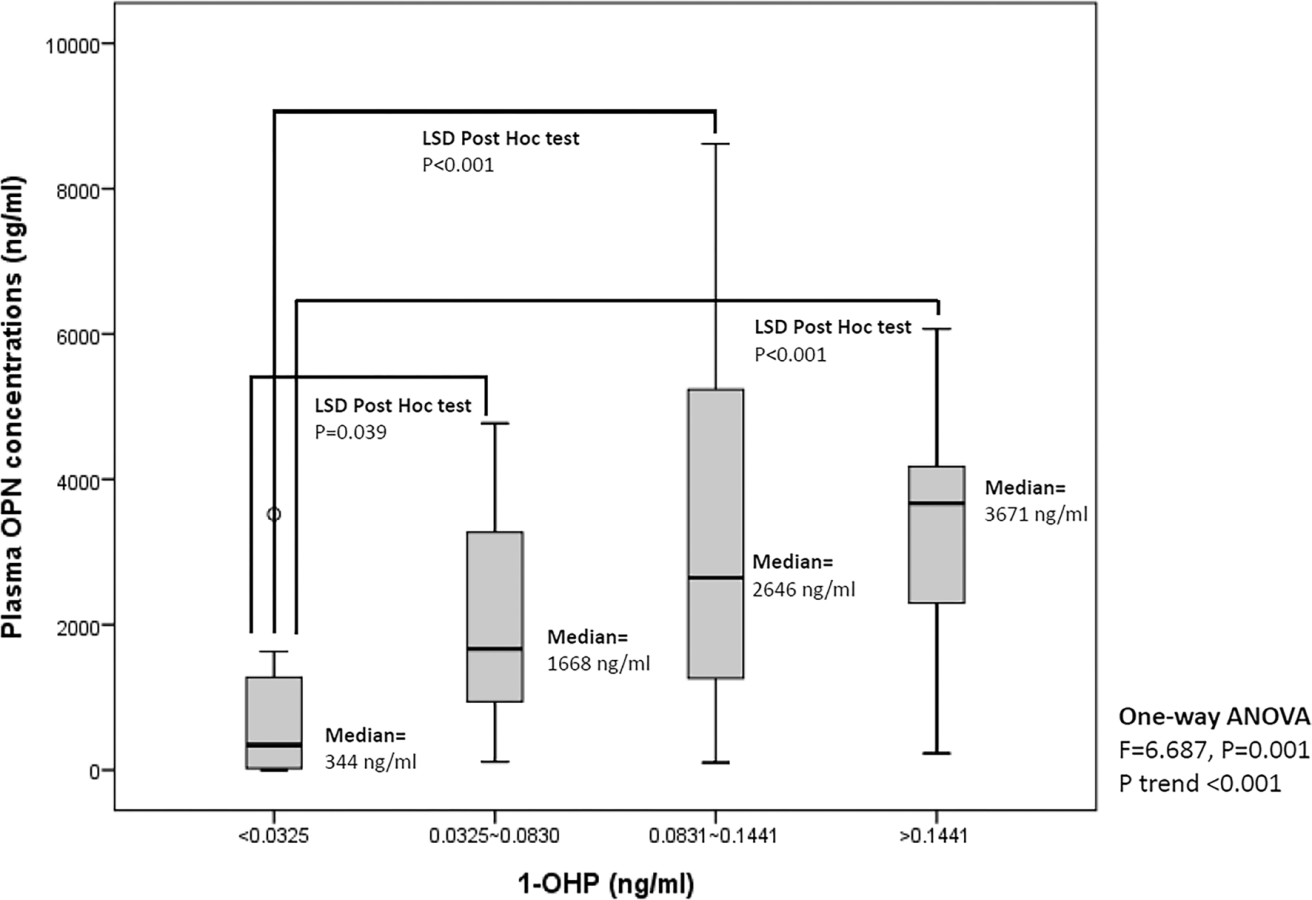
Plasma OPN concentrations were correlated with urinary 1-OHP concentration in a human population. Fasting blood samples and urine samples were collected from nonsmoking young adults for analysis. The plot shows plasma OPN concentrations across quartiles of urinary 1-OHP levels. One-way ANOVA and P trend analysis were used to assess the differences and trends in OPN levels. Fisher’s least significance difference (LSD) method was used in post hoc comparisons to test the difference in OPN levels for a two-by-two comparison with the 1-OHP groups

Figure 7 summarize the levels of urinary 1-OHP and OPN concentrations among different 1-OHP groups. The results revealed that the participants across the quartiles of urinary 1-OHP levels had higher average OPN levels (F = 6.687, *p* = 0.001; *p* for trend < 0.001). We selected the predictive variables—age, sex, BMI, waist circumference, time spent indoors, alcohol consumption, vitamin supplement intake, and regular exercise habit—in the linear regression model by using a stepwise approach (Table 3). Regardless of whether the urinary 1-OHP level was considered a continuous (model 1: Log1-OHP) or categorical (model 2: 1-OHP; ≥ 0.083 ng/mL vs. < 0.083 ng/mL) variable, it was associated with increased OPN levels (as a continuous variable: B = 0.88, 95% confidence interval [CI] = 0.42–1.35, *p* < 0.001; as a categorical variable: B = 0.43, 95% CI = 0.02–0.84, *p* = 0.041). The creatinine-adjusted 1-OHPG levels was associated with increased OPN levels (model 3: B = 1.86, 95% confidence interval [CI] = 0.30–3.42, *p* = 0.02). Additional file 1: Table 5 presents a moderate correlation between 1-OHP (as a PAH metabolite) and PM_2.5_, with the correlation coefficient between PM_2.5_ and 1-OHP of 0.458 and *p* < 0.001. Considering the correlation coefficients between PM_2.5_ or 1-OHP and OPN, 1-OHP was more relevant to OPN than to PM_2.5_. Collectively, these results support the assumption that OPN is an early subclinical biomarker of PAH exposure.

**Table 3:** The relationships between LogOPN and 1-OHP in non-smoking subjects used for linear regression models

	Univariate analysis				Model 1 (stepwise)^a^				Model 2 (stepwise)^b^				Model 3 (stepwise)^c^			
	B	95%CI		*p*-value	B	95%CI		*p*-value	B	95%CI		*p*-value	B	95%CI		*p*-value
Log1-OHP	1.11	0.64	1.57	< 0.001	0.88	0.42	1.35	< 0.001								
1-OHP (≥ 0.083 ng/ml vs. < 0.083 ng/ml)	0.69	0.27	1.10	0.002					0.43	0.02	0.84	0.041				
1-OHP(µg/g creatinine)	2.18	0.05	3.86	0.012									1.86	0.30	3.42	0.020
Age (year)	− 0.09	− 0.16	− 0.02	0.009												
BMI (Kg/m^2^)	0.04	− 0.02	0.11	0.163												
Waist circumstances (cm)	0.01	− 0.01	0.04	0.297												
Time spent indoors (hours)	− 0.06	− 0.12	0.004	0.065					− 0.05	− 0.10	0.01	0.07				
Time spent outdoors (hours)	0.07	− 0.02	0.15	0.118												
Transit time (hours)	0.12	− 0.24	0.47	0.504												
Gender (Male vs. Female)	0.44	− 0.01	0.88	0.053	0.34	− 0.02	0.71	0.063	0.40	0.02	0.78	0.041				
Alcohol drinking (Yes vs. No)	0.03	− 0.32	0.38	0.859												
Vitamin supplement (Yes vs. No)	− 0.92	− 1.40	− 0.43	< 0.001	− 0.53	− 1.00	− 0.07	0.025	− 0.65	− 1.13	− 0.16	0.010	− 0.62	− 1.00	− 0.24	0.002
Medicine taken (Yes vs. No)	− 0.84	− 1.83	0.16	0.098												
Regular exercise (Yes vs. No)	− 0.09	− 0.38	0.21	0.545												

^a^The predictive variables including Log1-OHP, age, BMI, waist circumstances, time spent indoors, gender, alcohol drinking, vitamin supplement, and regular exercise

^b^The predictive variables including 1-OHP (median categories), age, BMI, waist circumstances, time spent indoors, gender, alcohol drinking, vitamin supplement, and regular exercise

^c^The predictive variables including 1-OHP(µg/g creatinine), age, BMI, waist circumstances, time spent indoors, gender, alcohol drinking, vitamin supplement, and regular exercise

## Discussion

Many PAH species have been widely detected and quantified in ambient PM_2.5_; however, their contribution to the risk of ambient PM_2.5_-associated vascular diseases is largely unknown. By using VSMCs as an in vitro model for vascular toxicity, the ToxCast database, and ambient concentrations of PM_2.5_-bound PAHs, we identified that BkF is the PAH species in PM_2.5_ that contributes the highest to the risk of vascular toxicity after PM_2.5_ exposure. BaP-TEQs derived from the AhR activation potency may be used to assess the vascular toxicity risk of a PAH mixture. Furthermore, PM_2.5_ induced OPN based on AhR activation in in vitro and in vivo. Because plasma OPN concentrations were correlated with PM_2.5_ exposure as well as the concentrations of the urinary PAH metabolite 1-OHP in humans, OPN may be used as a vascular toxicity biomarker for PM_2.5_-bound PAH exposure in epidemiological studies in the future.

PAHs, which are produced by the incomplete combustion of organic materials, such as fossil fuels and cigarette smoke, are widely distributed in the environment. Some epidemiological studies have suggested an association between PAH exposure and the risk of cardiovascular diseases [46–49]. However, the association between PM_2.5_-bound PAHs and cardiovascular diseases has rarely been reported. Recently, Xu et al. [50] reported that PM_2.5_-bound PAHs are associated with elevated diastolic blood pressure in healthy adults. Most high-molecular-weight airborne PAHs, such as BbF, BkF, BaP, BeP, and DBA, are found in ambient PM, and those with a low molecular weight were found in the gas phase. Keebaugh et al. [51] demonstrated that concentrated ultrafine ambient particles (CAPs) induced arterial plaques in apoE gene-deleted mice. However, when high molecular weight PAHs were removed from CAP by thermal denuding (deCAP), exposure to deCAP failed induce the lesions, suggesting that high-molecular-weight PAHs in PM play a role in the early development of atherosclerosis. Consistently, we demonstrated that some high-molecular-weight PAHs in PM_2.5_, including BbF, BkF, BaP, and DBA, increased the VSMC migration ability and may contribute greatly to PM_2.5_-induced vascular toxicities.

Chronic exposure to BaP accelerated the atherosclerosis process in apoE-knockout mice [52]. Kerley-Hamilton et al. [53] demonstrated that BaP induced atherosclerosis to a greater extent in mice with the high-affinity AhR than in those with the low-affinity AhR. Thus, AhR activation might be involved in the BaP-accelerated development of atherosclerosis. The present study and previous studies [54, 55] have demonstrated that exposure to ambient PM induced AhR-mediated gene expression and inflammatory cytokines in VSMCs, endothelial cells, and macrophages, which play a role in the development of vascular dieases. Some studies have suggested that AhR activation mediates inflammatory responses, which may promote the development of vascular diseases [55–57]. We observed that AhR activation was not only involved in ambient PM_2.5_-induced proinflammatory cytokine secretion in VSMCs but also influenced the migration ability of VSMCs. The organic extract of PM_2.5_ containing PAHs caused similar biological effects in VSMCs. Therefore, we concluded that PM_2.5_-bound PAHs play a role in PM_2.5_-induced vascular toxicities through AhR activation.

In the present study, we demonstrated that PM_2.5_-bound PAHs enhanced the VSMC migration ability based on their AhR activation potencies. The data from ToxCast obtained using the CALUX assay and from Vondrsacek’s study using the AZ-AhR assay [36] revealed that BbF, BkF, and D(a,h)A were more potent AhR agonists than BaP in the human cell line HepG2, in the order of BkF > D(a,h)A > BbF. The present total BaP-TEQs estimated using REPs derived from both data sources and by considering the PAH levels measured in PM_2.5_ indicated that BkF was the most dominant contributor to the overall AhR-mediated activity, followed by BbF and D(a,h)A. The combination of BkF and BbF contributed to more than 86% of AhR activation by PM_2.5_-bound PAHs. Consistently, a recent study [58] reported that the combination of BkF and BbF contributed the most to the total BaP-TEQs based on the CALUX-based REPs. The substantially similar findings in the present study obtained using two data sources (U.S. EPA’s ToxCast and Vondrsacek’s study) support the application of the determination of AhR-mediated activity using in vitro human cell-based bioassays in the risk assessment of PAH mixtures. Moreover, the ToxCast database analysis revealed that BkF and BbF activated the NF-κB reporter assay (Additional file 1: Table 2). Considering that NF-κB activation also mediated VSMC migration and IL-6 secretion, BkF and BbF may be the most important PAHs in PM_2.5_ contributing to vascular toxicities.

**Fig. 8:**
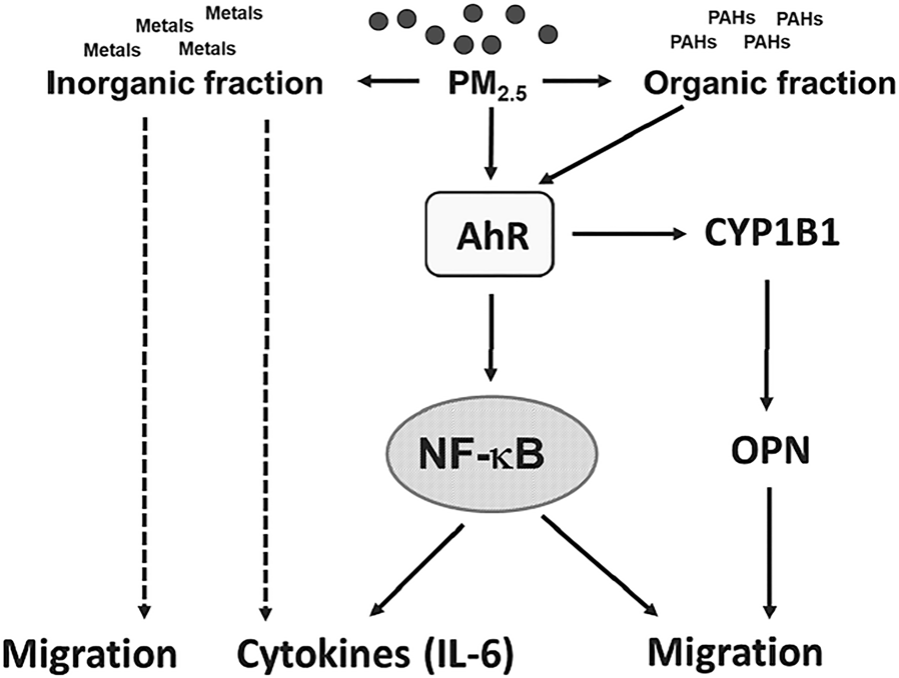
Summary of the mechanisms for PM_2.5_-induced VSMC migration in the present study

Exposure to ambient PM_2.5_ not only significantly increased OPN secretion in VSMCs and in mice but was also correlated with elevated plasma OPN levels in healthy humans [9]. We demonstrated that ambient PM_2.5_ increased OPN secretion in an AhR-dependent manner in VSMCs (Fig. 8). However, the promoter of OPN has no AhR response element [26]. CYP1B1, transcriptionally inducible by AhR activation, is expressed in cardiovascular tissues and contributes to the development of hypertension and neointimal growth caused by vascular injury [43, 59]. In the present study, we reported that CYP1B1 was involved in PM_2.5_-induced VSMC migration and OPN expression. AhR activation may have partially mediated OPN expression via CYP1B1 after PM_2.5_ exposure (Fig. 8). Notably, CYP1B1 was not involved in PM_2.5_-induced IL-6 expression. CYP1B1 mediated angiotensin II or PDGF-BB-induced VSMC proliferation and migration via arachidonic acid metabolites or reactive oxygen species, respectively [43, 59]. However, how CYP1B1 mediated PM_2.5_-induced VMSC migration and OPN expression remains unclear. High-molecular-weight PAHs are the major components of PM_2.5_ responsible for AhR activation and increased ONP secretion. 1-OHP is a metabolite of high-molecular-weight PAHs, and its urinary concentration has been used to assess the effects of exposure to PAH mixtures [17, 38, 60]. OPN is considered a prognosis biomarker for vascular diseases [61]. The correlation of urinary 1-OHP and plasma OPN levels in human participants suggests that OPN may be used in human studies to identify individuals at risk of vascular diseases after PAH or PM_2.5_ exposure in the future.

Although the inorganic extract of PM_2.5_ failed to increase *CYP1B1* or *OPN* expression, the inorganic extract moderately increased the migration ability of VSMCs (Figs. 3A and 8). It is likely that PAHs may not be the sole constituents contributing to enhanced cell migration. Previously, we demonstrated that PM increased the migration ability of VSMCs via not only the AhR dependent pathway but also the NOX1/ROS-dependent NF-κB signaling pathway [7]. We considered both organic fraction (PAHs) and inorganic extracts (metals) are constituents contributing to migration in VSMCs. The inorganic extract (metals) might increase the migration ability of VSMCs via the NOX1/ROS-dependent NF-κB signaling pathway.

In a preliminary study, we evaluated effects of PM_2.5_ on multiple cytokines secretion in BALF of mice. We found that only IL-6, CXCL-1 and RANTES levels were significantly elevated (data not shown). Our previous study [7]showed that PM_2.5_ increased IL-6 and CXCL-1 in a NOX1/ROS-dependent NF-κB pathway. IL-6 is a cytokine involved in vascular inflammation [62]. Exposure to PM_2.5_ immediately induced pulmonary inflammation and increased IL-6 secretion, but both these effects gradually decreased after continued exposure to PM_2.5_ for 4–8 weeks [7]. Our present results revealed that PM_2.5_ induced IL-6 expression through the NF-kB pathway, which was downstream of the AhR activation pathway (Figure 8). Although the inorganic extract of PM_2.5_ did not contain PAHs and failed to activate AhR, it induced IL-6 expression (Figure 8). The inorganic extract contains various metals (Additional file 1: Table 4). Yeh et al [63] demonstrated that vanadium pentoxide induced IL-6 production in VSMCs and promoted VSMC migration and proliferation. Thus, both organic and inorganic components in PM_2.5_ appear to induce IL-6 expression. The metals in PM_2.5_ might induce IL-6 expression through AhR-independent mechanisms.

There are some limitations in our present study. In our in vitro studies, we did not identify which chemical components in PM_2.5_ are responsible for IL-6 induction in VSMCs. The potential interaction between chemical components in PM_2.5_ on AhR activation was not explored either. In the animal study with the low AhR activity mice, we cannot exclude the possibility that AhR activation in other cell types, such as endothelial cells and macrophages, also involved in the mechanism of OPN induction by PM_2.5_. The main limitation of the human study was the small number of subjects, which may not be representative of the overall population distribution. Secondly, the results cannot be extrapolated to older age groups and specific disease groups as this study subjects were young health people. Thirdly, the exposure assessment of PM_2.5_ for individuals is to assess the short-term exposure of 24 h and further observation may be required for long-term effects.

## Conclusions

Our present study offered evidences that PAHs in PM_2.5_ play a role in PM_2.5_-induced vascular toxicities via the AhR dependent mechanisms, although their concentrations in PM_2.5_ were relatively low. Based on AhR activation potencies, we identified that BkF contributed to the most AhR agonistic activity of PM_2.5_, and suggested that the BaP-TEQ approach may be applied for assessing the vascular toxicity risk of PAH mixtures. Furthermore, OPN may be used as a vascular toxicity biomarker for PAH mixtures exposure.

## Supplementary Information

**Additional file 1**. The supplementary information of PM_2.5_ and PM_2.5_-related results of in vitro, animal and human studies.

## Data Availability

All the data presented in this study are included in the article.

## Abbreviations

AhR: Aryl hydrocarbon receptor
B6.D2: B6.D2NAhrd/J
BALF: Bronchoalveolar lavage fluid
BaA: Benz(a)anthracene
BaP: Benzo(a)pyrene
BbF: Benzo(b)fluoranthene
BeP: Benzo(e)pyrene
BkF: Benzo(k)fluoranthene
CAPs: Concentrated ultrafine ambient particles
CYP1A1: Cytochrome P450 1A1
CYP1B1: Cytochrome P450 1B1
C: Concentrations
D(a,h)A: Dibenz(a,h)anthracene
deCAP: CAP by thermal denuding
d_2_H_2_O: Double-distilled water
ELISA: Enzyme-linked immunosorbent assay
FBS: Fetal bovine serum
H&E: Hematoxylin and eosin
HTS: High-throughput screening
IL-6: Interleukin-6
LSD: Fisher’s least significant difference
NHRI: National Health Research Institutes
1-OHP: 1-Hydroxy pyrene
OPN: Osteopontin
PM_2.5_: Fine particulate matter
PAHs: Polycyclic aromatic hydrocarbons
PDGF: Platelet-derived growth factor
qPCR: Quantitative polymerase chain reaction
REP: Relative potencies
RPFs: Relative potency factors
TEQ: Toxic equivalent
TMB: (E)-2,3',4,5'-tetramethoxystilbene
VSMCs: Vascular smooth muscle cells
