# Supplementary material for: Aryl hydrocarbon receptor activation-mediated vascular toxicity of ambient fine particulate matter: contribution of polycyclic aromatic hydrocarbons and osteopontin as a biomarker
Source: Part Fibre Toxicol. 2022 Jun 23;19:43. doi: 10.1186/s12989-022-00482-x (PMC9219152; doi:10.1186/s12989-022-00482-x)
Supplement: Supplementary file 1 — Additional file 1. The supplementary information of PM2.5 and PM2.5-related results of in vitro, animal and human studies. [file 12989_2022_482_MOESM1_ESM.docx]

**A.**

**PM_2.5_ Organic Inorganic**

**B.**

**PM_2.5_ Organic Inorganic**

**Figure S1. Effects of organic and inorganic extracts of PM_2.5_ on OPN and IL-6 expression in VSMCs.**

VSMCs were treated with d_2_H_2_O, DMSO, 25 μg/mL PM_2.5_, organic or inorganic extract of PM_2.5_ for 48 h. The following parameters were determined: (A) *OPN* relative mRNA levels; (B) *IL-6* relative mRNA levels. The results are presented as mean ± standard deviation (SD) for one experiment with 3 replicates. Control: d_2_H_2_O or DMSO treatment as control.

**B.**

**A.**

**PM_2.5_ Organic Inorganic**

**PM_2.5_ Organic Inorganic**

**D.**

**C.**

**PM_2.5_ Organic Inorganic**

**PM_2.5_ Organic Inorganic**

**Figure S2. Effects of 12.5, 25 and 50 μg/ml PM_2.5_, organic or inorganic extracts of PM_2.5_ on gene expression in VSMCs.**

VSMCs were treated with d_2_H_2_O, DMSO, 12.5, 25 and 50 μg/mL PM_2.5_, organic or inorganic extract of PM_2.5_ for 48 h. The following parameters were determined: (A) *OPN* relative mRNA levels; (B) *IL-6* relative mRNA levels; (C) *CYP1A1* relative mRNA levels; (D) *CYP1B1* relative mRNA levels. The results are presented as mean ± SD for two independent experiments with 3 replicates. Control: d_2_H_2_O or DMSO treatment as control.

**B.**

**A.**

*****

*****

*****

*****

*****

**#**

**#**

*****

**PM_2.5_ Inorganic**

**PM_2.5_ Inorganic**

**D.**

**C.**

*****

*****

*****

*****

*****

*****

*****

**#**

**#**

*****

**PM_2.5_ Inorganic**

**PM_2.5_ Inorganic**

**Figure S3. Effects of PM_2.5_ or organic extracts with of PM_2.5_ on OPN and IL-6 expression in reducing CYP1B1 or OPN expression-VSMCs.**

VSMCs were treated with d_2_H_2_O, DMSO, 25 μg/mL PM_2.5_, organic extract of PM_2.5_ with or without 0.1 μM TMB for 48 h. The following parameters were determined: (A) *OPN* relative mRNA levels; (B) *IL-6* relative mRNA levels. The results are presented as mean ± SD for three independent experiments with 2 or 3 replicates. **p* < 0.05, compared with d_2_H_2_O -treated cells (n=3, one-way ANOVA). #*p* < 0.05, compared with CYP1B1 inhibitor–treated cells (n=3, two-way ANOVA). Control: d_2_H_2_O treatment as control. NC-VSMCs and siOPN-VSMCs were treated with d_2_H_2_O, DMSO, 25 μg/mL PM_2.5_, organic extract of PM_2.5_ for 48 h. The following parameters were determined: (C) *OPN* relative mRNA levels; (D) *IL-6* relative mRNA levels. The results are presented as mean ± SD for three independent experiments with 2 or 3 replicates. **p* < 0.05, compared with NC-control -treated cells (n=3, one-way ANOVA). #*p* < 0.05, compared with NC-PM_2.5_ treatment –treated cells (n=3, two-way ANOVA). NC: negative control, siOPN: OPN siRNA, Control: d_2_H_2_O or DMSO treatment as control.

**B.**

**A.**

**C.**

*****

**D.**

*****

**Figure S4. Effects of fiberglass filter materials on cytotoxicity and gene expression in VSMCs and mice.**

VSMCs were treated with d_2_H_2_O and fiberglass filter materials for 48 h. The following parameters were determined: (A) MTT assay; (B) *OPN, IL-6 and CYP1B1* relative mRNA levels. The results are presented as the mean ± SD for the one experiment. The C57BL/6J mice were aspirated with d_2_H_2_O, fiberglass filter materials and 25 μg of PM_2.5_ twice weekly for 8 weeks. (C) Total cell numbers of BALF, (D) OPN protein in BALF. Each value represents the mean ± SD of eight mice. ^*^P < 0.05 for comparison with the d_2_H_2_O -treated mice (n=8, one-way ANOVA)

**Table S1. Ambient concentrations of seven PAHs in PM_2.5_ in the period of January to March 2018**

(Unit: ng/m^3^)

| Month/Day  PAHs species | 01/19 | 01/22 | 01/25 | 01/28 | 01/31 | 02/02 | 02/06 | 02/08 | 02/11 | 03/07 | Mean | SD | Median |
| --- | --- | --- | --- | --- | --- | --- | --- | --- | --- | --- | --- | --- | --- |
| Benz(a)anthracene | 0.09 | 0.05 | 0.07 | 0.07 | 0.09 | 0.06 | 0.06 | 0.08 | 0.06 | 0.10 | 0.07 | 0.02 | 0.07 |
| Benzo(b)fluoranthene | 0.40 | 0.19 | 0.29 | 0.23 | 0.37 | 0.26 | 0.28 | 0.33 | 0.23 | 0.41 | 0.30 | 0.08 | 0.28 |
| Benzo(k)fluoranthene | 0.16 | 0.08 | 0.11 | 0.09 | 0.17 | 0.10 | 0.11 | 0.13 | 0.09 | 0.17 | 0.12 | 0.03 | 0.11 |
| Benzo(a)pyrene | 0.18 | 0.08 | 0.15 | 0.11 | 0.15 | 0.10 | 0.12 | 0.16 | 0.10 | 0.15 | 0.13 | 0.03 | 0.14 |
| Dibenz(a,h)anthracene | 0.05 | 0.02 | 0.04 | 0.02 | 0.04 | 0.02 | 0.02 | 0.04 | 0.02 | 0.03 | 0.03 | 0.01 | 0.03 |
| Benzo(e)pyrene | 0.35 | 0.16 | 0.24 | 0.13 | 0.21 | 0.19 | 0.21 | 0.28 | 0.18 | 0.21 | 0.22 | 0.06 | 0.21 |
| Chrysene | 0.26 | 0.13 | 0.18 | 0.17 | 0.17 | 0.16 | 0.21 | 0.20 | 0.18 | 0.26 | 0.19 | 0.04 | 0.18 |

**Table S2. The AC_50_ and AC_10_ values of AhR and NF-κB reporter activities for individual PAHs in the ToxCast Database.**

| PAHs | Toxcast  AhR AC_50_  (μM) | Toxcast  AhR AC_10_  (μM) | Toxcast  NF-κB AC_50_  (μM) |
| --- | --- | --- | --- |
| Naphthalene | inactive | - | inactive |
| Acenaphthylene | 91.5 | 69.18 | inactive |
| Acenaphthene | inactive | - | inactive |
| Fluorene | inactive | - | inactive |
| Phenanthrene | inactive | - | inactive |
| Anthracene | inactive | - | inactive |
| Fluoranthene | inactive | - | inactive |
| Pyrene | inactive | - | inactive |
| Cyclopenta(c,d)pyrene | -^a^ | - | -^a^ |
| Benz(a)anthracene | 9.31 | 1.10 | inactive |
| Chrysene | 7.86 | 1.51 | inactive |
| Benzo(b)fluoranthene | 0.498 | 0.06 | 31.70 |
| Benzo(k)fluoranthene | 0.01 | 0.00 | 1.33 |
| Benzo(e)pyrene | 1.34 | 0.22 | inactive |
| Benzo(a)pyrene | 2.71 | 0.26 | inactive |
| Perylene | -^a^ | - | -^a^ |
| Indeno(1,2,3,-cd)pyrene | -^a^ | - | -^a^ |
| Dibenz(a,h)anthracene | 0.05 | 0.02 | inactive |
| Benzo(b)chrysene | -^a^ | - | -^a^ |
| Benzo(g,h,i)peryene | inactive | 0.14 | inactive |
| Coronene | -^a^ | - | -^a^ |
| Dibenzo(a,e)pyrene | -^a^ | - | -^a^ |

^a^Not available.

**Table S3. Mass concentrations of PAHs in organic and inorganic extracts of PM_2.5_**

| PAHs species | PAHs in  PM_2.5_  (ng/mg) | | PAHs in  organic extracts  (ng/mg) | | PAHs in inorganic  extracts | |
| --- | --- | --- | --- | --- | --- | --- |
| Napthalene | 0.019 | 0.016 | |  | |  |
| Acenapthylene | 0.072 | 0.164 | |  | |  |
| Acenapthene | 0.062 | 0.054 | |  | |  |
| Fluorene | 0.027 | 0.024 | |  | |  |
| Phenanthrene | 0.215 | 0.195 | |  | |  |
| Anthracene | 0.088 | 0.086 | |  | |  |
| Fluoranthene | 0.800 | 1.056 | |  | |  |
| Pyrene | 0.687 | 0.511 | |  | |  |
| Cyclopenta(c,d)pyrene | 1.203 | 1.213 | |  | |  |
| Benzo(a)anthracene | 0.689 | 0.664 | | Lower than  Limit of Detection | |  |
| Chrysene | 1.897 | 2.111 | |  | |  |
| Benzo(b)fluoranthrene | 4.642 | 4.951 | |  | |  |
| Benzo(k)fluoranthrene | 1.884 | 1.871 | |  | |  |
| Benzo(e)pyrene | 3.854 | 2.752 | |  | |  |
| Benzo(a)pyrene | 1.679 | 1.391 | |  | |  |
| Perylene | 0.325 | 0.427 | |  | |  |
| Indeno(1,2,3,-cd)pyrene | 3.614 | 3.518 | |  | |  |
| Dibenz(a,h)anthracene | 0.571 | 0.206 | |  | |  |
| Benzo(b)chrysene | 0.234 | 0.262 | |  | |  |
| Benzo(g,h,i)peryene | 6.134 | 4.523 | |  | |  |
| Coronene | 3.215 | 2.058 | |  | |  |
| Dibenzo(a,e)pyrene | 0.247 | 0.204 | |  | |  |
| ΣPAHs | 32.160 | 28.256 | |  | |  |

**Table S4. Mass concentrations of metals in organic and inorganic extracts of PM_2.5_**

| Metals species | Metals in  PM_2.5_  (μg/mg) | Metals in  organic extracts  (μg/mg) | Metals in  inorganic extracts  (μg/mg) |
| --- | --- | --- | --- |
| Ti | 0.147 | 0.000 | 0.056 |
| V | 0.287 | 0.001 | 0.429 |
| Cr | 0.442 | 0.000 | 0.049 |
| Mn | 0.341 | 0.000 | 0.411 |
| Fe | 2.994 | 0.000 | 1.047 |
| Co | 0.005 | 0.000 | 0.005 |
| Ni | 0.106 | 0.000 | 0.109 |
| Cu | 0.174 | 0.009 | 0.158 |
| Zn | 3.939 | 0.366 | 1.382 |
| As | 0.029 | 0.005 | 0.019 |
| Se | 0.045 | 0.004 | 0.038 |
| Sr | 0.074 | 0.000 | 0.026 |
| Mo | 0.070 | 0.001 | 0.088 |
| Cd | 0.008 | 0.001 | 0.005 |
| Sb | 0.031 | 0.002 | 0.019 |
| Ba | 3.495 | 0.043 | 0.092 |
| La | 0.003 | 0.000 | 0.000 |
| Ce | 0.003 | 0.000 | 0.001 |
| Pr | 0.000 | 0.000 | 0.000 |
| Nd | 0.001 | 0.000 | 0.000 |
| Sm | 0.000 | 0.000 | 0.000 |
| Yb | 0.000 | 0.000 | 0.000 |
| Lu | 0.000 | 0.000 | 0.000 |
| Pt | 0.000 | 0.000 | 0.000 |
| Pb | 0.223 | 0.004 | 0.063 |
| U | 0.000 | 0.000 | 0.000 |

**Table S5. Correlation coefficient between OPN, PM_2.5_ and 1-OHP in non-smoking subjects used for spearman's rank correlation**

|  | OPN (ng/ml) |  | PM_2.5_ (μg/m^3^) |  | 1-OHP (ng/ml) |
| --- | --- | --- | --- | --- | --- |
| OPN (ng/ml) | 1.000 |  |  |  |  |
| PM_2.5_ (μg/m^3^) | 0.426 | ^**^ | 1.000 |  |  |
| 1-OHP (ng/ml) | 0.534 | ^**^ | 0.458 | ^**^ | 1.000 |
| ** p<0.001 | | | | | |
